# Augmented Stability in Leaving Original Internal Fixation with Multidimensional Cross Locking Plate through Mini‐Open Femoral Anterior Approach for Aseptic Femoral Shaft Nonunion: A Retrospective Cohort Study

**DOI:** 10.1111/os.13581

**Published:** 2022-11-21

**Authors:** Taoguang Wu, Wei Zhang, Zuhao Chang, Zhengguo Zhu, Lijun Sun, Peifu Tang, Hua Chen

**Affiliations:** ^1^ Department of Orthopedic Surgery the fourth medical center, Chinese PLA General hospital Beijing China; ^2^ School of Sports Engineering Beijing Sport University Beijing China

**Keywords:** Augmentation plate, Femoral shaft nonunion, Femoral anterior approach, Lateral locking plate, Multidimensional cross locking plate

## Abstract

**Objective:**

Aseptic femoral shaft nonunion constitutes approximately 1%–10% of all femoral shaft fractures treated with intramedullary nail (IMN) fixation, possibly attributable to the lack of anti‐rotational stability. Although a lateral locking plate (LP) with retainment of original IMN has shown the most success, lateral LP inflicts significant surgical trauma on patients. Therefore, the Multidimensional Cross Locking Plate (MDC‐LP) was designed based on a mini‐open femoral anterior approach. We aim to report and compare the technical aspects and clinical outcomes of using anterior MDC‐LP or lateral LP with retention of original IMN for the treatment of aseptic femoral shaft nonunion.

**Methods:**

In this single center retrospective cohort study, records of 49 patients who had undergone revision of femoral shaft aseptic nonunion with anterior MDC‐LP or lateral LP while retaining the original IMN from January 2015 to October 2019 were retrospectively reviewed. Information on patients' demographics, clinical data, and surgical outcomes were gathered and analyzed. X‐ray and CT scans were used for bone union evaluation and the lower extremity functional scale (LEFS) was used for follow‐up functional evaluation. For quantitative data, the Student's *t*‐test was used if the data were normally distributed. The Mann–Whitney *U*‐test was used for non‐normally distributed data. For qualitative data, the Chi‐square test was used for comparisons.

**Results:**

Twenty‐seven patients were treated with anterior MDC‐LP, and 22 patients were treated with lateral LP. There are no significant differences in age, sex, BMI, time since initial femoral shaft fracture, initial fracture type (close/open), nonunion type, or nonunion location between patients' group. Among patients treated with anterior MDC‐LP, an average of 2‐months advantage in time to union was observed (4.09 months *vs.* 6.8 months in the lateral LP group: *P* = 0.000), smaller incision was required for MDC‐LP installment (7.7 cm *vs* 17.1 cm in lateral LP group: *P* = 0.000).

**Conclusions:**

For the treatment of aseptic femoral shaft nonunion with retainment of original IMN, anterior MDC‐LP *via* mini‐open femoral anterior approach described in this study is a better option than lateral LP for achieving faster bone union and satisfactory functional outcome with less surgical trauma.

## Graphical abstract

Muti‐dimensional cross locking plate (MDC‐LP) was positioned on anterior surface of the femur, designed to be anatomically compatible with anterior surface and anterior curve of the femur. MDC‐LP provides multidimensional cross stability *via* bi‐cortical screw fixation. In sagittal plane, proximal locking screws are pointed proximally and distal locking screws distally. In coronal plane, locking screws are drilled at a 30° divergent angle to clamp original IMN. MDC‐LP provides anti‐rotational stability through more bi‐cortical screw fixation. In addition, bi‐cortical screw fixation decreases the width of the medullary cavity away from the isthmic areas, which resists the displacing muscular forces of adductor muscle.

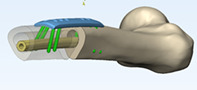

## Introduction

Intramedullary nail (IMN) fixation is the gold standard for the treatment of fresh femoral shaft fractures. Despite incidences of aseptic femoral shaft nonunion having decreased significantly, it occurs from time to time, constituting 1%–10% of all cases of femoral shaft fractures, possibly due to the lack of anti‐rotational stability of IMN.^1^, ^2^, ^3^, ^4^, ^5^, ^6^ How to treat femoral shaft nonunion remains challenging for orthopedic trauma surgeons.

Current treatments for aseptic femoral shaft nonunion include nail dynamization, exchanging nail, double plates, the Ilizarov technique, and augmentation plate (AP) with retainment of original IMN. Due to the variable bone healing rates achieved by current treatments,^7^, ^8^ the optimal treatment for aseptic femoral shaft nonunion is debated. Although nail dynamization is a simple and safe procedure, the treatment could cause leg length discrepancies, especially among patients with comminuted fractures.^9^, ^10^ Exchanging nail is preferred by many surgeons, but the treatment yields variable healing rates due to potential heat injury or insufficient anti‐rotational stability.^11^, ^12^, ^13^, ^14^, ^15^ Double plates could obtain high union rates due to its rigid stability that promotes the integration of the bone grafting, but placement of the double plate causes extensive soft tissue stripping at the nonunion site.^16^, ^17^ In comparison, the Ilizarov technique is a minimally invasive procedure, but the construction of an external fixator increases the risk for pin tract infections and decreases patients' quality of life.^18^

**Fig. 1:**
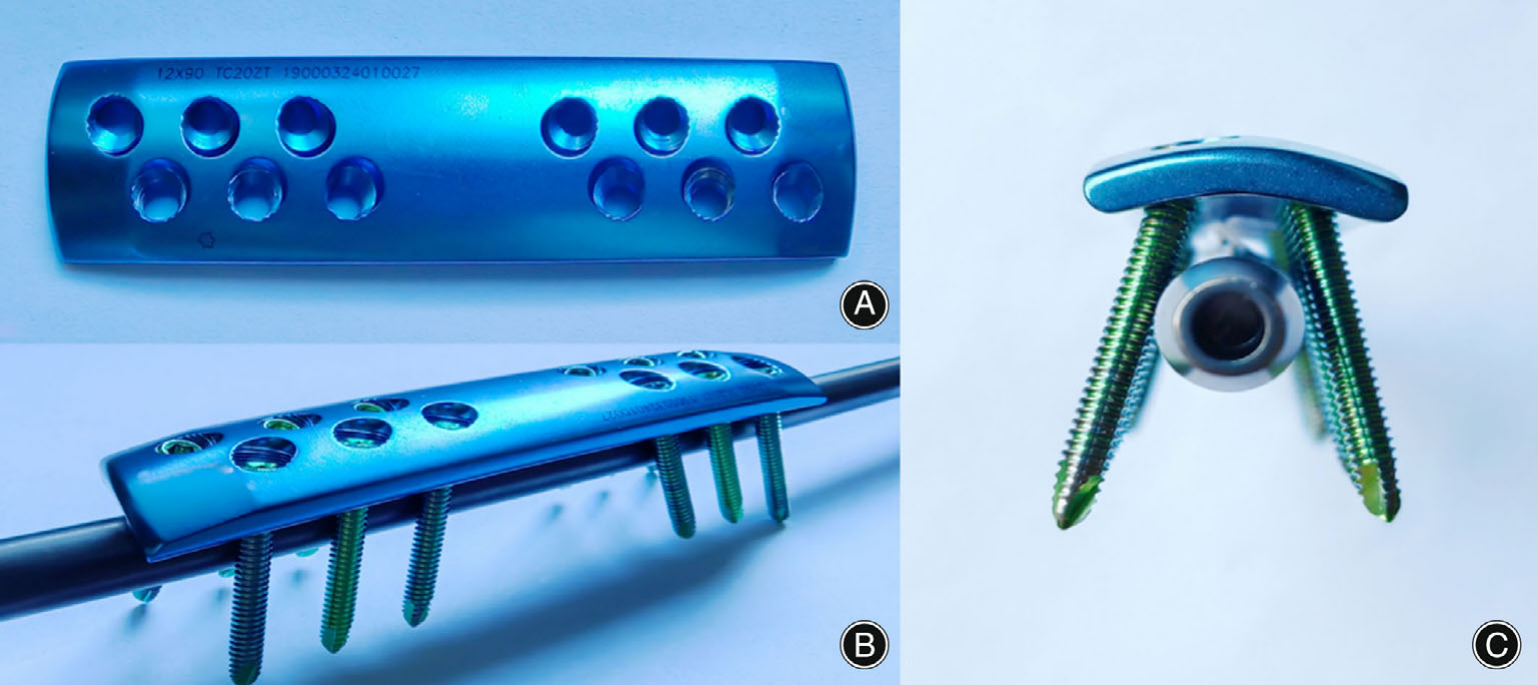
Multidimensional cross locking plate (MDC‐LP) with bi‐cortical screw fixation while retaining original intramedullary nail (IMN). (A) Top view of the MDC‐LP. (B) Lateral view of MDC‐LP with bi‐cortical screw fixation while retaining original IMN installed on femoral shaft. (C) Coronal view of MDC‐LP with bi‐cortical screw fixation at a 30° divergent angle in the coronal plane clamping the original IMN on femoral shaft

AP with retainment of original IMN is a promising treatment for femoral shaft nonunion. The classic method, lateral locking plate (LP) proposed by Ueng *et al*., leaves original IMN *in situ*, performs bone grafting to repair bone defects, and applies the AP plate to the fracture site through a lateral or post lateral approach to counter rotational instability.^1^, ^19^, ^20^, ^21^, ^22^, ^23^, ^24^ Although this technique has achieved success in treating femoral shaft nonunion after IMN and with broken IMN,^19^ previous literature reports that a lateral or posterolateral approach incision of at least 17 cm is required to fully expose the nonunion site, especially in the medial side of the fracture site, for removal of fibrous tissue and autogenous bone grafting, creating massive destruction of soft tissue.^20^, ^25^ Moreover, due to the anterior femoral curvature and obstruction from the original IMN, bi‐cortical screw fixation cannot be achieved in lateral LP. To enhance anti‐rotational stability, lateral LP places a longer plate at the fracture site to enable installment of more uni‐cortical screws away from the fracture site to adapt the original IMN, which makes the procedure more invasive. To solve this problem, we designed the Multidimensional Cross Locking Plate (MDC‐LP)^26^ (Tianjin Zhengtian Medical Instrument Co, Ltd., Authorized Patent Number: CN 201621144111.0, JP 6751995B2) to be placed at the nonunion site through a mini‐open femoral anterior approach (Fig. 1).

*Via* a mini‐open femoral anterior approach, a surgical window that can shift proximally or distally when flexing or extending the hip/knee joint is established to enable the placement of a plate longer than the incision to minimize incision length and soft tissue destruction. Since MDC‐LP is placed at the anterior surface of the femur, MDC‐LP was designed to be anatomically compatible with the anterior surface and anterior curve of the femur to eliminate stimulus to soft tissue. Moreover, MDC‐LP provides multidimensional stability of the nonunion site *via* bi‐cortical screw fixation, where the locking screws are drilled at a 30‐degree divergent angle in the coronal plane to clamp the original IMN. The proximal locking screws are pointed proximally and the distal locking screws distally in sagittal plane, which allow screws to be placed through a small incision. Biomechanical tests^26^ show that MDC‐LP provides more anti‐rotational stability than the classic lateral LP proposed by Ueng *et al*.

The aim of this study was: (i) to investigate the clinical outcome; (ii) to evaluate the technical advantage; and (iii) to compare the option of the approach using MDC‐LP through a mini‐open femoral anterior approach for the treatment of aseptic femoral shaft nonunion with retention of original IMN.

## Patients and Methods

### Patients

We retrospectively reviewed the records of 49 patients who had undergone revision of femoral shaft aseptic nonunion with AP while retaining the original IMN from January 2015 to October 2019 for this single center retrospective cohort study. They were divided into two groups: anterior MDC‐LP group and lateral LP group. During the follow‐up period, all the patients completed all regular serial assessments, without any sequential loss of follow‐up. The study was approved by the medical ethics committee of PLA General Hospital (S2020‐005‐01) and was registered in the Chinese Clinical Trial Registry (ChiCTR‐ORC‐17012547). All patients provided informed consent. This work has been reported in line with the STROCSS criteria.^27^

Inclusion criteria included: (i) femoral shaft aseptic nonunion; (ii) radiographic evidence of nonunion or no radiographic signs of progress in healing in last three consecutive months; (iii) age ≥ 18‐years old; and (iv) bone defect ≤3 cm after debridement.

Exclusion criteria included:(i) femoral malalignment: angulation greater than 5° in either coronal plane or sagittal plane, or malrotation greater than 15°; (ii) Infectious nonunion; (iii) patients complicated with nervous system diseases involving peripheral motor nerves; (iv) initially compound fractures; (v) nonunion in pathological fracture; and (vi) patients with mental illness or other diseases who could not comply with the treatment protocol.

### Procedures

Operative treatment was exclusively performed by two of our authors, who are experienced with treatment of femoral shaft nonunion. In both groups, the patient was placed in a supine position on radiolucent operative table. Intravenous prophylactic antibiotics were administered to all patients. The entire leg was prepared, including the iliac crest.

#### Anterior MDC‐LP Technique

**Fig. 2:**
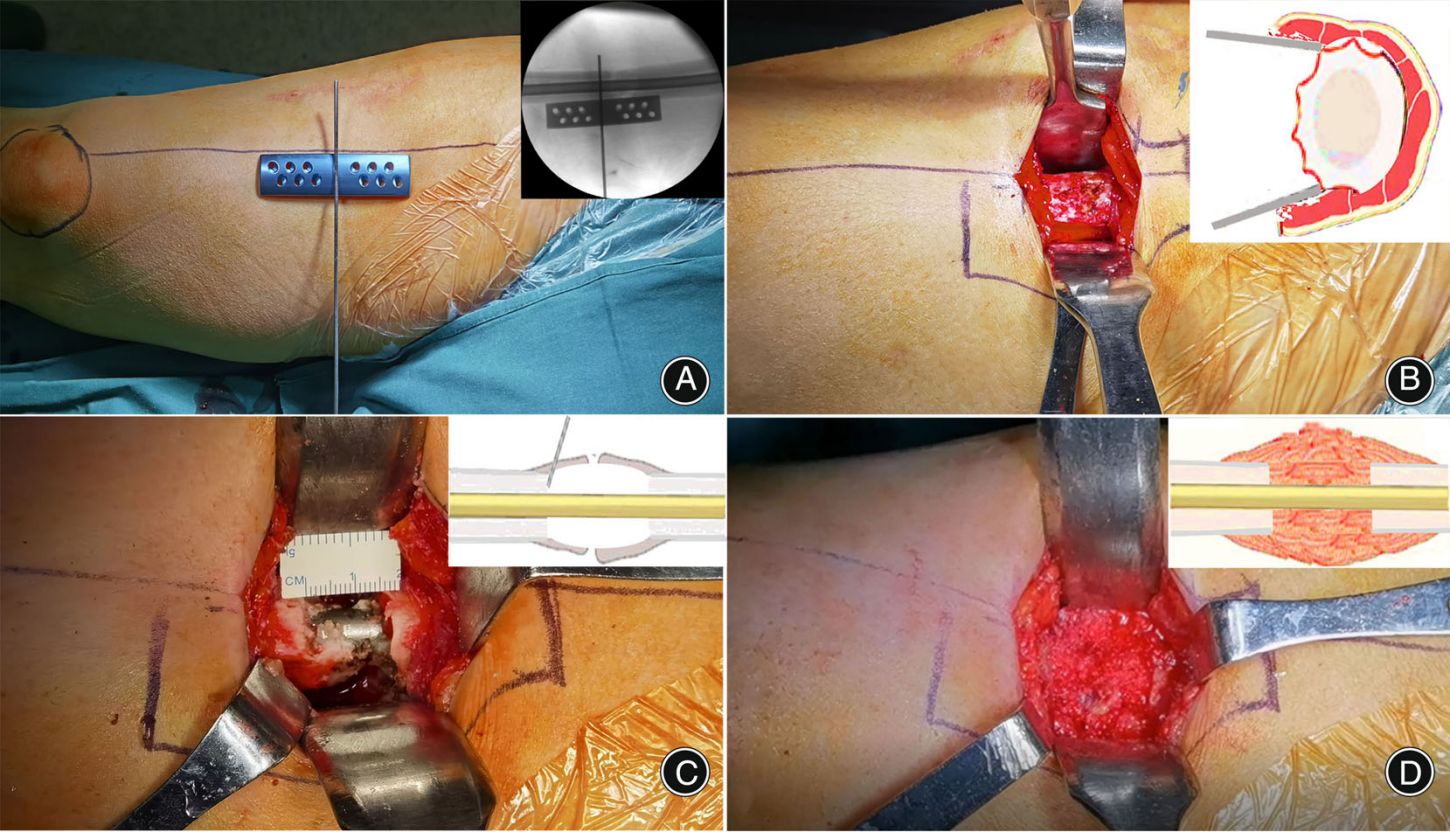
The debridement at the nonunion site. (A) A longitudinal line was marked between anterior superior iliac spine (ASIS) and patella. Multidimensional cross locking plate (MDC‐LP) was placed and centered on the nonunion site under the guidance of fluoroscopy. (B) The fracture ends were decorticated using a sharp osteotome for atrophic nonunion. (C) Fibrous tissue and sclerotic bone at the nonunion site were debrided until bleeding can be observed at the fracture end to cause bone defect at the nonunion site. (D) Cancellous bone grafts taken from the iliac spine was used to fill the cavity

For patients in MDC‐LP group, the femoral anterior approach was used.^28^ A longitudinal line was marked between the anterior superior iliac spine (ASIS) and the patella (Fig. 2A). An incision was made centered on the nonunion site. The skin, subcutaneous tissue, and fascia were sequentially incised. The vastus intermedius was exposed through the lateral intermuscular space between vastus lateralis and rectus femoris or the medial intermuscular space between vastus medialis and rectus femoris. Anterior aspect of the femur was exposed by sharply incising the vastus intermedius along the longitudinal axis of the femur. Subsequently, vastus intermedius was subperiosteally elevated laterally or medially.

For atrophic nonunion, the fracture ends were decorticated subperiosteally using an osteotome (Fig. 2B). Fibrous tissue and sclerotic bone at the nonunion site were debrided until bleeding was observed at the fracture end causing bone defect at the nonunion site (Fig. 2C). Cancellous bone grafts taken from the iliac spine were used to fill the cavity (Fig. 2D). For hypertrophic nonunion, the bone was flattened with osteotomes before placing the MDC‐LP.

**Fig. 3:**
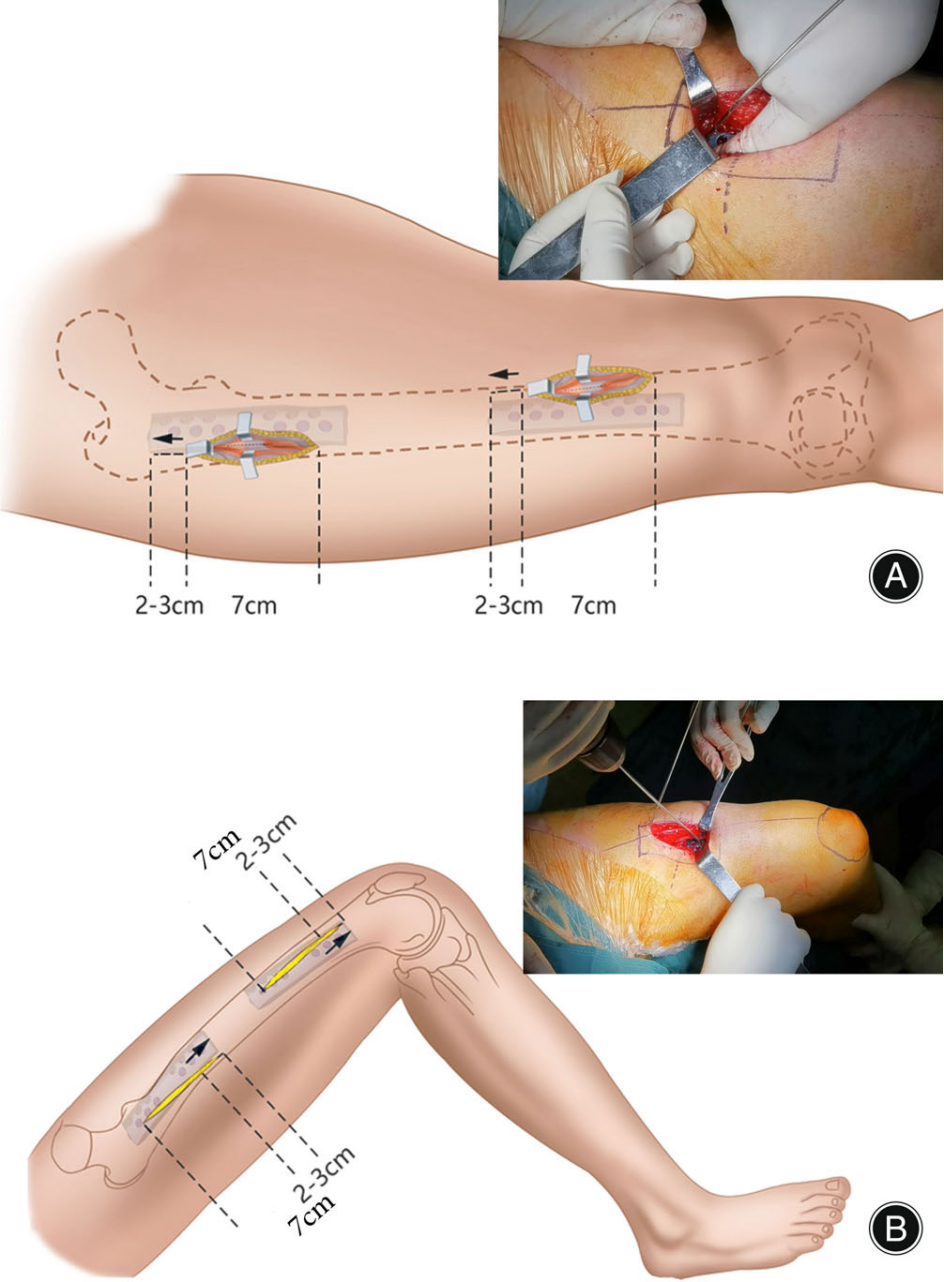
Placement of multidimensional cross locking plate (MDC‐LP) and bi‐cortical screw fixation through mini‐open femoral anterior approach. (A) When knee and hip are placed in an extended position to relax the quadricep femoris, retracting the muscle proximally to shift the incision 2–3 cm proximally inserts the proximal screws. (B) When knee and hip are placed in a flexion position to stress the quadriceps femoris, the surgical window automatically shifts 2–3 cm distally to insert the distal screws

Subsequently, the appropriate MDC‐LP (shown in Video 1) designed to be anatomically compatible with the anterior surface and anterior curve of the femur was positioned on the anterior surface of the femur. Knee and hip were placed in an extended position to relax the quadricep femoris. By retracting the muscle proximally to shift the incision 2–3 cm proximally, proximal screws were inserted (Fig. 3A). To insert the distal screws, knee and hip were placed in a flexion position to stress the quadriceps femoris, which automatically shifts the surgical window 2–3 cm distally (Fig. 3D). A suction drain was placed after suturing the periosteum and fascia. The incision was closed layer by layer.

For patients with broken IMN or at risk for broken IMN due to more than 14 months after initial IMN fixation, the original IMN was exchanged with an unreamed intact nail of the same size. All other steps remained the same.

#### Lateral LP Technique

For patients in lateral LP group, an appropriate 3.5 mm locking plate (Synthes, Paoli, PA, USA) (shown in Video 2) was placed and secured at the nonunion site *via* a femoral lateral approach.^1^, ^24^ Debridement of the nonunion site, bone grafting, and closure of the incision were the same as those of MDC‐LP group.

On postoperative day one, patients were allowed to perform full‐range motion of the knee and hip without weight‐bearing in bed to avoid knee or hip stiffness. Isometric contraction and straight leg‐raising on the quadricep femoris were exercised to prevent muscle atrophy. Depending on the condition of fracture healing, weight‐bearing of the affected limb was gradually carried out.

### Evaluation and Follow‐up

Bone union was determined radiographically and clinically independent of the operating surgeon. Radiographic union was determined by bridging bone on three out of four cortices without a radiolucent line at the nonunion site according to X‐ray or bridging bone constituting more than 25% of cross‐sectional area according to CT.^29^ Clinical bone union was determined by minimal or no pain when the patient bears weight on the affected lower limb.^30^

**Fig. 4:**
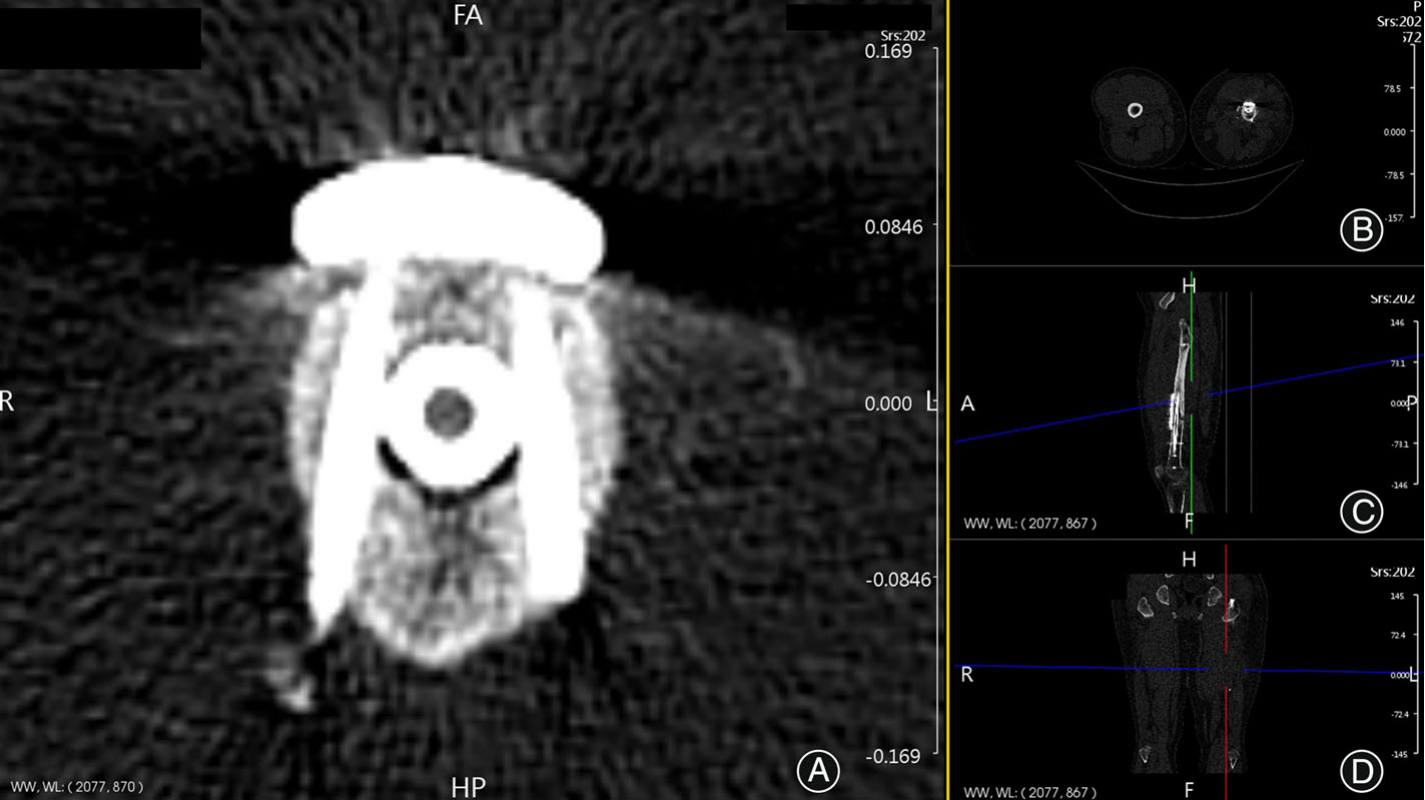
Three‐dimensional CT reconstruction of multidimensional cross locking plate (MDC‐LP) bi‐cortical screw fixation with original intramedullary nail (IMN) during postoperative follow‐up. (A) Coronal section of MDC‐LP with bi‐cortical screw fixation clamping the original IMN on femoral shaft during 3D reconstruction. (B) Transverse scanning image during 3D reconstruction. (C) Sagittal section image during 3D reconstruction. (D) Coronal section image during 3D reconstruction

Recovery of the affected limb was assessed at the last follow‐up using the lower extremity functional scale (LEFS).^31^ Moreover, patients were evaluated using the following parameters: (i) length of the MDC‐LP or LP; (ii) incision length for plate placement in centimeters; (iii) number of bi‐cortical screws determined using three‐dimensional (3D) CT reconstruction (Fig. 4); and (iv) complications including implant failure, wound problems, neurovascular injury, infection, fracture malunion and nonunion, reoperation, and re‐fracture.

### Statistical Analysis

SPSS statistical software version 20.0 (IBM, Chicago, IL, USA) was used for statistical analysis. For quantitative data, the student's *t*‐test was used for comparisons between groups if the data were normally distributed. The Mann–Whitney *U*‐test was used for non‐normally distributed data. For qualitative data, the chi‐square test was used for comparisons between groups. The significance level was set at α = 0.05.

## Results

### Patients

Patients treated with MDC‐LP or lateral LP for femoral shaft nonunion had similar demographics (Table 1). Moreover, there were no significant differences in time since initial fracture, initial fracture type (close/open), AO/OTA classification, number of previous operations, nonunion type, and nonunion location between patients treated with MDC‐LP or lateral LP for femoral shaft nonunion (Table 1).

**TABLE 1:** Patient demographic

Variable	MDC‐LP group (n = 27)	Lateral‐LP group (n = 22)	*P* value
Age	36.74 ± 11.37	34.50 ± 12.42	0.514
Sex (male/female)	19/8	14/8	0.761
BMI (kg/m^2^)	26.05 ± 2.53	25.26 ± 4.44	0.464
Time since injury (months)	26.7 ± 35.13	17.18 ± 11.27	0.229
Initial fracture type (close/open)	25/2	20/2	0.495
32‐A	15	13	0.657
32‐B	10	6	
32‐C	2	3	
Previous operation times	1.59 ± 1.19	1.15 ± 1.14	0.889
Nonunion type^20^			
Atrophic	15	13	1
Hypertrophic	12	9	
Nonunion location^21^			
Isthmic	12	9	0.969
Infra‐isthmic	8	7	
Supra‐isthmic	7	6	
Follow‐up (months)	32.2 ± 11.6	27.7 ± 10.7	0.176

*Note*: The values are given as the mean and standard deviation, except for sex, nonunion type, and location.

Abbreviations: BMI, body mass index; lateral LP, lateral locking plate; MDC‐LP, Multidimensional Cross Locking Plate.

### Procedure Parameters

**Fig. 5:**
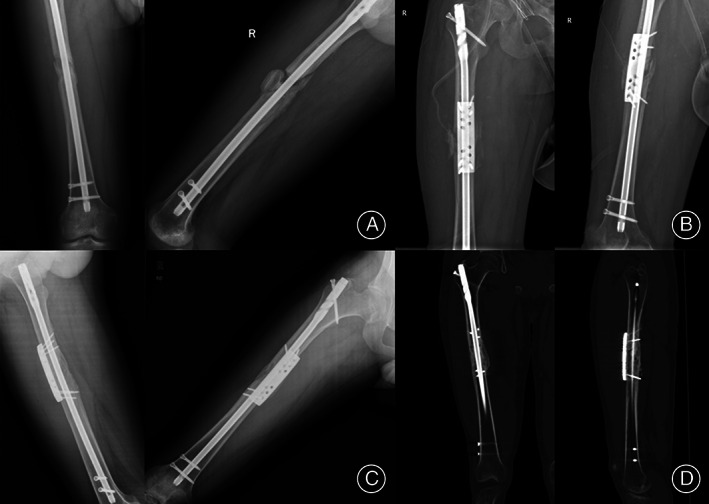
Twenty‐seven‐year‐old male with right isthmal femoral shaft nonunion after intramedullary nail (IMN) underwent revision with anterior multidimensional cross locking plate (MDC‐LP) while retaining original IMN. (A) X‐ray showing no breakage of original IMN fixation and atrophic nonunion without obvious deformity. (B) Immediate post‐operative X‐ray after revision with anterior MDC‐LP while retaining original IMN. (C) X‐ray of anterior–posterior and lateral views showing bone union 4 months postoperatively. (D) 3D coronal and sagittal sections showing fracture healing 4 months postoperatively

Post‐operative finding reveals significant differences in time to union, number of bi‐cortical screws, blood transfusion volume during hospitalization, length of incision for exposing nonunion site, and length of plate placed at fracture site between patients treated with MDC‐LP or lateral LP (Table 2). At 6 months postoperatively, all patients treated with MDC‐LP achieved bone union, but only 63.6% of patients treated with lateral LP achieved bone union (*P* = 0.001). At 9 months postoperatively, 91% of patients treated with lateral LP achieved bone union (*P* = 0.449). Time to union was on average 2 months shorter among patients treated with MDC‐LP (4.09 months *vs* 6.8 months in the lateral LP group: *P* = 0.000) (Fig. 5).

**TABLE 2:** Clinical data

Variable	MDC‐LP group (n = 27)	Lateral‐LP group (n = 22)	*P* value
Bone union at 6 months^a^	27 (100%)	14 (63.6%)	0.001
Bone union at 9 months^a^	27 (100%)	21 (91%)	0.449
Time to union (months)^b^	4.09 ± 1.30	6.8 ± 1.60	0.000
Incision length (cm)^b^	7.67 ± 2.02	17.09 ± 6.73	0.000
Plate length (cm)^b^	9.70 ± 0.91	17.69 ± 4.98	0.000
The difference between plate and incision	2.04 ± 2.07	0.73 ± 4.39	0.178
Number of bicortical screws^b^	7.26 ± 1.40	2.05 ± 1.95	0.000
Blood transfusion volume during hospitalization (ml)^b^	222.59 ± 220.31	745.45 ± 478.25	0.000
LEFS at last follow‐up	72.93 ± 7.85	62.59 ± 10.06	0.000
Complications			
Superficial infection	0	5	0.000
Persistent nonunion	0	1	0.000

Abbreviations: MDC‐LP, multidimensional cross locking plate; lateral LP, lateral locking plate; LEFS, lower extremity functional scales.

^a^ The values are given as the number of the patients, with the percentage in parenthesis.

^b^ The values are given as the mean and standard deviation.

Intraoperative findings show that rotational instability of femoral shaft was found for all patients with aseptic femoral shaft nonunion. Treatment with MDC‐LP or lateral LP eliminated rotational instability. Nevertheless, shorter plates were used for patients treated with MDC‐LP (9.7 cm *vs* 17.7 cm in Lateral LP group: *P* = 0.000), and shorter incisions were used for patients treated with MDC‐LP (7.7 cm *vs* 17.1 cm in Lateral LP group: *P* = 0.000). Placement of MDC‐LP on the fracture site *via* an anterior approach required an incision that is 3 cm shorter on average than the length of the plate (*P* = 0.000). Whereas placement of LP *via* a lateral approach required an incision that is 1 cm longer on average than the length of LP (*P* = 0.000). More bi‐cortical screws were used in MDC‐LP (7 *vs* 2 in the lateral LP group: *P* = 0.000). Less blood was transfused during hospitalization for patients treated with MDC‐LP (222 ml *vs* 745 ml in Lateral LP group: *P* = 0.000).

### Functional Outcomes

The follow‐up period of all patients ranges from 12 to 48 months with an average of 30 months (30.0 ± 11.2 months). All patients completed self‐reported outcome scales LEFS. Better functional outcome LEFS scores were observed in the patients treated with MDC‐LP (73 *vs* 62 in Lateral LP group: *P* = 0.000).

### Complications

No complications were observed among patients treated with MDC‐LP. Among patients treated with lateral LP, five patients suffered from superficial infections that were managed successfully by local treatment alone. One patient had persistent nonunion 9 months after lateral LP, so this patient underwent revision using MDC‐LP *via* a femoral anterior approach with retainment of original IMN and LP. This patient achieved bone union 5 months after MDC‐LP and 26 months after initial femoral shaft fracture. At the last follow‐up 12 months post‐final revision, the patient achieved a satisfactory functional outcome measured by a LEFS score of 80.

## Discussion

We observed that anterior MDC‐LP obtained a satisfactory functional outcome with faster bone healing and less surgical trauma for the treatment of aseptic femoral shaft nonunion with retention of original IMN. The newly designed MDC‐LP is a shorter plate that not only provides more bi‐cortical screw stability but also clamps the original IMN to enhance stability, and promoting the integration of bone grafting. A femoral anterior approach through the intermuscular space by a small incision creates a flexible surgical window to complete the operation, which avoids excessive dissection of soft tissue, protects the blood supply to nonunion site, and promotes bone healing.

### Clinical Outcome

Anterior MDC‐LP technique not only accelerates fracture healing, but also does not affect the function of the affected limb. Previous studies showed that lateral LP has higher union rate of 96%, with average time for fracture healing of 5.9–9 months.^15^, ^32^, ^33^, ^34^ Mittal *et al*.^24^ used lateral LP to treat 21 patients with femoral shaft nonunion, the average time for fracture healing was 6 months ranging from 4 months to 8 months. Uliana *et al*.^23^ used lateral LP to treat 22 patients with femoral shaft nonunion in a multicenter study, the average time for fracture healing was 11.7 months ranging from 2 months to 16 months. In contrast, the time for fracture healing in the patients included in this study was obviously shorter after anterior MD‐LP, with an average of 4.09 months (an average of 6.8 months after lateral LP). The LEFS outcome tool revealed that better functional outcome of 73 ± 7.9 was achieved in anterior MDC‐LP (63.0 ± 10.1 in lateral LP).

### Augmented Stability of Original IMN

**Fig. 6:**
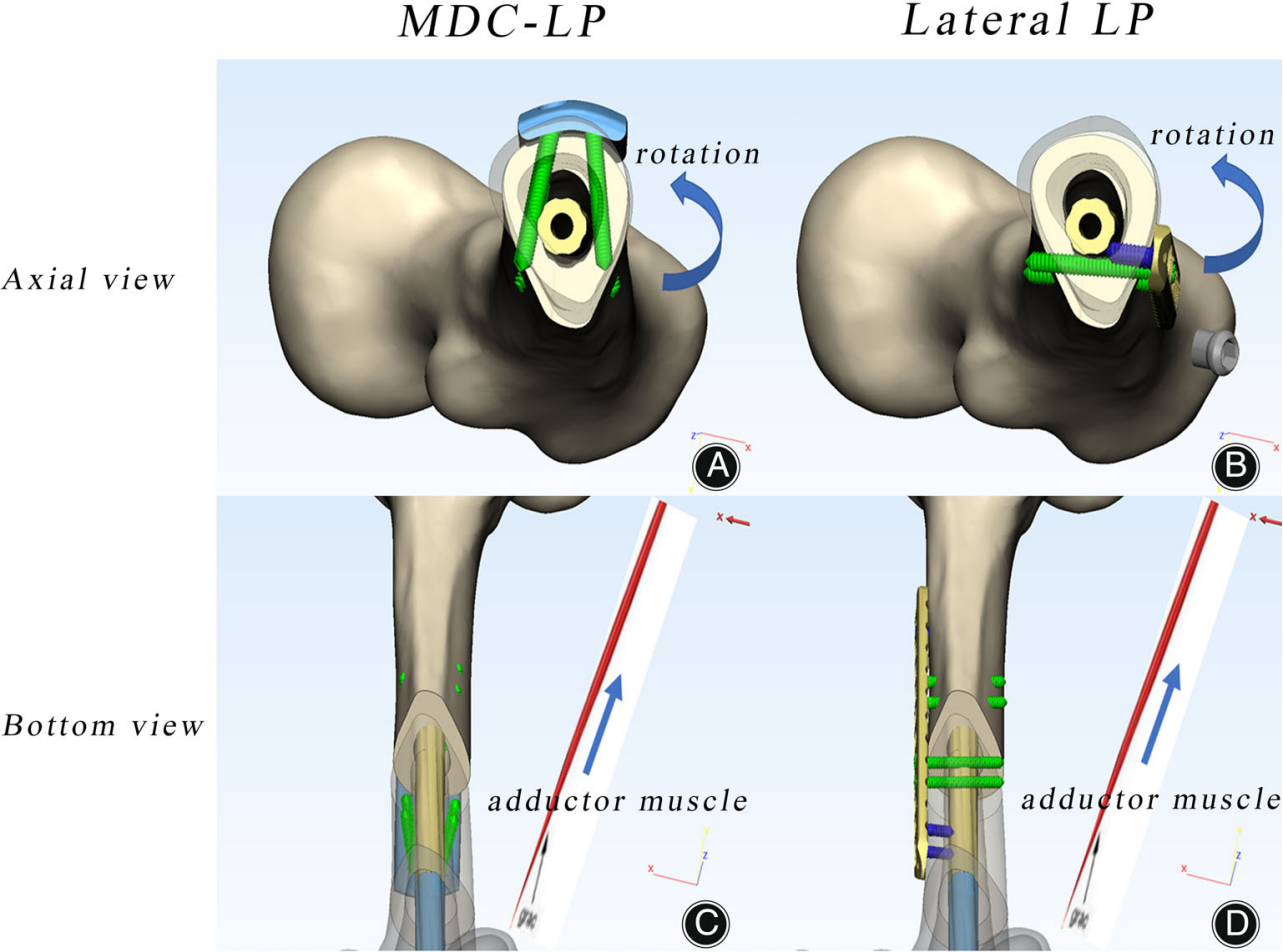
Schematic diagram showing how multidimensional cross locking plate (MDC‐LP) and lateral locking plate (LP) promote anti‐rotational stability and resist instability due to widening of medullary canal from displacing forces of adductor muscle. The MDC‐LP with bi‐cortical screw fixation is located at anterior aspect of the femur, and the lateral LP with uni‐cortical screws is at lateral aspect of the femur. Both are closely attached to the femur and achieve similar function. Bi‐cortical screws are labeled in green, uni‐cortical screws in blue, MDC‐LP in blue and LP in yellow. Axial view (A, B), bottom view (C, D). (A) MDC‐LP under rotational force; (B) lateral LP under rotational force; (C) MDC‐LP under adductor muscle force; (D) lateral LP under adductor muscle force

MDC‐LP effectively supplements anti‐rotational stability to the original IMN fixation. It is well known that aseptic femoral nonunion is attributable to insecure fixation of IMN and lack of anti‐rotational stability.^1^, ^24^ Therefore, enhancing anti‐rotational stability at the fracture site is crucial for addressing hypertrophic and atrophic nonunion, especially for atrophic nonunion. After debridement of fibrotic and sclerotic tissue at the atrophic nonunion site, bone cortical defect will be formed, which will eliminate contact between fracture ends and further reduce the anti‐rotational stability of the interlocked nailing construct. Lateral LP enhances anti‐rotational stability by placing a longer plate at the fracture site to enable installment of more uni‐cortical screws away from the fracture site to adapt the original IMN (Fig. 6A). Although this construct provides sufficient anti‐rotational stability,^35^ the lateral LP construct could be optimized by substituting uni‐cortical screws for bi‐cortical screws to supply additional anti‐rotational stability. MDC‐LP is designed to enable bi‐cortical screw fixation where the locking screws are drilled at a 30° divergent angle in the coronal plane to clamp the original IMN (Fig. 6B). The proximal locking screws are pointed proximally and the distal locking screws distally in sagittal plane, which allow screws to be placed through a small incision. Clinical data reveals that whereas lateral LP uses bi‐cortical screws (2.05 ± 1.95), MDC‐LP uses more bi‐cortical screws (7.26 ± 1.4) for promoting anti‐rotational stability. Biomechanical studies indicate a 6.1‐fold increase in torsional stiffness with anterior MDC‐LP (6.16 ± 0.06 N·m/deg) compared with lateral LP (1.02 ± 0.04 N·m/deg) in rotational instability model of femoral shaft nonunion with IMN.^26^

Moreover, post‐operative CT evidence of patients treated with anterior MDC‐LP reveals that bi‐cortical screws tightly clamp the IMN (Fig. 4). Thus, we have reasons to believe that bi‐cortical screw fixation in anterior MDC‐LP decreases the width of the medullary cavity away from the isthmic areas to resist the displacing muscular forces of adductor muscle (Fig. 6C). On the other hand, lateral LP is missing screw block from medial side of the femur (Fig. 6D). Future biomechanical studies are necessary for assessing such benefits of bi‐cortical screw fixation in anterior MDC‐LP.

### Option of Surgical Approach

In addition to enhancing anti‐rotational stability to promote bone union, MDC‐LP can be placed at the nonunion site *via* a mini‐open femoral anterior approach through the intramuscular space, which has many biological advantages for the exposure of the nonunion site compared to lateral LP. Since MDC‐LP is placed at the anterior surface of the femur, MDC‐LP was designed to be anatomically compatible with the anterior surface and anterior curve of the femur to eliminate stimulus to soft tissue (Fig. 1). Second, *via* a mini‐open femoral anterior approach, we found that an anterior incision length of 6.8 cm in average not only thoroughly exposed the medial side of the nonunion site for removal of fibrous or sclerotic tissue and addition of autologous bone grafts, but also enabled the placement of MDC‐LP and fixation of bi‐cortical screws with an average plate length of 9.70 cm, which minimizes incision length for plate placement. However, according to previous literature and intraoperative findings in this study, an incision of at least 17 cm is required for placement of lateral LP, which causes massive soft tissue stripping and negatively affects functional outcomes.^20^ Finally, intraoperative findings reveal that the surgical window established by the mini‐open femoral anterior approach can shift proximally or distally when flexing or extending the hip/knee joint. The flexibility enabled by the mini‐open femoral anterior approach allows the placement of MDC‐LP on fracture site *via* an incision that is 3 cm shorter on average than the length of the plate. Moreover, we optimized the location of the mini‐open approach for exposing the nonunion site through the intermuscular space for MDC‐LP to minimize peeling and destruction of surrounding soft tissue.^28^ Considering that major motor nerve branches innervate the vastus lateralis through the lateral intermuscular space of the distal two‐thirds of femoral shaft, we took the mini‐open femoral anterior approach through the medial or lateral intermuscular space of the rectus femoris. If the nonunion site is located at the proximal one third of the femoral shaft, the lateral intermuscular space of the rectus femoris should be taken for the mini‐open femoral anterior approach. If the nonunion site is located at the distal two‐thirds of the femoral shaft, mini‐open femoral anterior approach should be taken through the medial intermuscular space of the rectus femoris to expose the nonunion site. In comparison, lateral LP adopted a lateral or posterolateral approach through the tensor fascia lata to expose the medial side of the nonunion site. A long incision of 17 cm on average through the entire intermuscular space is required to install LP that is 1 cm shorter on average than the incision length, which would contribute to poor cosmetic outcomes and increased risks of infection.^20^, ^25^

### Strengths and Limitations

Overall, in this single center retrospective cohort study, we demonstrated that patients treated with anterior MDC‐LP had shorter bone union time, less invasive procedures, and better functional outcomes than those treated with lateral LP. We believe that the success of anterior MDC‐LP in treating aseptic femoral shaft nonunion is attributable to the enhanced anti‐rotational stability provided by additional bi‐cortical screw fixation and mini‐open femoral anterior approach to plate installment, which minimizes soft tissue dissection and maximizes protection of blood supply around the nonunion site to promote fracture healing.

Nevertheless, our study has its shortcomings. Considering that our study is a retrospective study, a future prospective cohort study is necessary for definitively verifying the efficacy and advantages of anterior MDC‐LP for treating aseptic femoral shaft nonunion. Also, mini‐open femoral anterior approach through the intermuscular space to expose the nonunion site requires sharp dissection of the vastus intermedius muscle, which could impair the sliding mechanism of the quadriceps femoris. Therefore, the effect of mini‐open femoral anterior approach on the sliding mechanism of the quadriceps femoris should be evaluated using more objective parameters, such as surface electromyography, isokinetic testing, and gait analysis in future studies.

### Conclusions

In this single center retrospective cohort study, we report and compare the technical aspects and clinical outcomes of using anterior MDC‐LP and lateral LP for the treatment of aseptic femoral shaft nonunion with retainment of original IMN. Based on analysis of clinical data, anterior MDC‐LP obtained a satisfactory functional outcome with faster bone healing and less surgical trauma compared to lateral LP. We believe that the better surgical outcome among patients treated with anterior MDC‐LP is attributable to enhanced anti‐rotational stability and biological environment by the following improved technical aspects. First, the newly designed MDC‐LP is a shorter plate that not only provides more bi‐cortical screw stability but also clamps the original IMN to enhance stability, promoting the integration of bone grafting. Second, a femoral anterior approach through the intermuscular space by a small incision creates a flexible surgical window to complete the operation, which avoids excessive dissection of soft tissue, protects the blood supply to nonunion site, and promotes bone healing. For the treatment of aseptic femoral shaft nonunion with retainment of original IMN, anterior MDC‐LP *via* mini‐open femoral anterior approach described in this study is a better option than lateral LP for achieving faster bone union and satisfactory functional outcome with less surgical trauma.

## Author Contributions

WTG, CH, and TF performed most of the investigation, data analysis, and wrote the manuscript; CH, SLJ, ZW, ZGZ, and CZH contributed to interpretation of the data and analyses. All of the authors have read and approved the manuscript.

## Supporting information


**Video 1:** Animation 1

**Video 2:** Animation 2
